# An Archaic Reference-Free Method to Jointly Infer Neanderthal and Denisovan Introgressed Segments in Modern Human Genomes

**DOI:** 10.1093/molbev/msag235

**Published:** 2026-09-17

**Authors:** Léo Planche, Anna Ilina, María C Ávila-Arcos, Flora Jay, Emilia Huerta-Sanchez, Vladimir Shchur

**Affiliations:** CNRS, INRIA, Laboratoire Interdisciplinaire des Sciences du Numérique, Université Paris-Saclay, Orsay 91400, France; International Laboratory of Statistical and Computational Genomics, Faculty of Computer Science, HSE University, Moscow, Russia; International Laboratory for Human Genome Research, Universidad Nacional Autónoma de México, Mexico; CNRS, INRIA, Laboratoire Interdisciplinaire des Sciences du Numérique, Université Paris-Saclay, Orsay 91400, France; The Smurfit Institute of Genetics, Trinity College Dublin, Dublin, Ireland; Department of Ecology, Evolution, and Organismal Biology, Brown University, Providence, RI, USA; International Laboratory of Statistical and Computational Genomics, Faculty of Computer Science, HSE University, Moscow, Russia

**Keywords:** local ancestry inference, Neanderthal, Denisovan, archaic introgression, admixture

## Abstract

Admixture between populations is a common feature of human history. Admixture events introduce new genetic variation that can fuel evolution. Characterizing the significance of admixture events on the evolution of populations across various species is of great interest to evolutionary geneticists. Local ancestry inference (LAI) methods infer genetic ancestry of an individual at a particular chromosomal location. Certain methods specialize in detecting archaic introgression, which consists of interbreeding between modern and archaic humans like Neanderthals and Denisovans. Most current LAI methods allow the detection of a single archaic ancestry, and post-processing may distinguish between multiple waves of introgression. These methods vary in how they choose archaic or modern reference genomes for the inference. Here, we present a new hidden Markov model-based method (DAIseg), which has the advantage of simultaneously distinguishing between multiple waves of ancient and recent admixture, using only modern human reference genomes. Simulations demonstrate that DAIseg achieves higher overall performance than state-of-the-art methods. We also apply DAIseg to Papuan populations to jointly detect Denisovan and Neanderthal introgressed segments, and identify a higher number of archaic segments than previous methods. Analysis of inferred introgressed segments shows that we can identify evidence for two Denisovan introgression events in Papuans. Overall, on top of being able to deal with both archaic and recent admixture, DAIseg provides a more principled approach for detecting and classifying Denisovan and Neanderthal segments which will improve downstream analysis of introgressed segments to infer the impact of archaic introgression in humans.

## Introduction

Throughout human history, admixture—the exchange of genes between isolated groups—has played a crucial role in shaping the genetic diversity observed in modern human populations (Nielsen et al. 2017; Narasimhan et al. 2019; Chintalapati et al. 2022; Tournebize et al. 2022). For instance, Neanderthals—identified through archeological findings—and Denisovans—discovered through sequencing a genome from a small finger bone—diverged from the lineage of anatomically modern humans (AMH) approximately 450,000 to 800,000 years ago, with varying estimates across studies (Green et al. 2006; Endicott et al. 2010; Meyer et al. 2016). Thanks to advances in genomic sequencing, we now have genetic material from both of these extinct archaic populations (Meyer et al. 2012; Prüfer et al. 2013, 2017; Mafessoni et al. 2020), enabling us to investigate their genetic relationships with AMH. Analyses of these ancient samples, alongside data from modern human populations, reveal that individuals of non-African origin carry approximately 2% to 3% Neanderthal DNA. Recent studies also report a lower prevalence (0.2%) of Neanderthal DNA in some African populations (Chen et al. 2020). Denisovan genetic ancestry is predominantly found in populations from Oceania, South and Eastern parts of Asia, and the Americas, with 3% to 4% Denisovan ancestry in Papuans (Reich et al. 2010; Witt et al. 2023).

Quantifying and studying archaic introgression can be approached in two main ways. The first, global ancestry inference, estimates the proportion of an individual’s genome derived from specific ancestries, such as Neanderthal or Denisovan. The second, local ancestry inference (LAI), focuses on identifying the specific genomic regions inherited from these archaic populations, which are the focus of this paper. Knowing where in the genome individuals carry archaic segments is helpful for providing critical insights into the role of natural selection acting on introgressed fragments (adaptive introgression), the timing of introgression events, the identification of introgression deserts in modern human genomes, the number of archaic sources and estimates of genetic diversity in archaic populations based on surviving fragments in modern genomes. Since only a limited number of archaic genomes have been sequenced, these fragments offer a unique opportunity to infer genetic diversity in archaic populations that interbred with AMH.

In LAI methods, chromosomes are conceptualized as mosaics of genomic tracts originating from multiple ancestral groups (Padhukasahasram 2014; Wu et al. 2021). LAI is a valuable tool for understanding population evolution, migration history, and disease risk (Padhukasahasram 2014; Fitak et al. 2018). Many LAI models rely mostly on hidden Markov models (HMMs), where hidden states represent ancestries and emissions correspond to observed haplotypes/genotypes (Baran et al. 2012; Hu et al. 2013; Guan 2014; Steinrücken et al. 2018). The ancient divergence between AMH and archaic populations results in substantial genetic distance, facilitating the detection of archaic ancestry in modern humans. However, the limited number of available archaic reference genomes may not fully capture the diversity of archaic individuals who actually contributed to modern humans, potentially underestimating archaic ancestry (Prüfer et al. 2013). Despite these challenges, various methods have been developed to detect archaic fragments in modern genomes using archaic reference genomes (Plagnol and Wall 2006; Browning et al. 2018; Skov et al. 2018; Durvasula and Sankararaman 2020). Alternative approaches, such as hmmix, identify archaic ancestry without using Neanderthal or Denisovan genomes, instead using a modern human population without introgression as an outgroup (eg African populations) (Skov et al. 2018). While hmmix enables the detection of archaic fragments, its application often requires multiple runs and/or extensive post-processing to distinguish Neanderthal from Denisovan ancestry, making comparisons across studies challenging (Ongaro and Huerta-Sánchez 2024). Classifying whether archaic segments come from Neanderthals or Denisovans is important because these segments are used to infer the time of introgression, the number of introgression events, and the genetic relationships between archaic populations.

Different studies have used slightly different approaches for classifying archaic segments. For example, to identify Denisovan ancestry in Papuans, the authors of Skov et al. (2018) first ran hmmix to identify archaic segments with Africans as an outgroup, followed by another run using Africans, Europeans, and East Asians as outgroups. The segments that are detected only in the first run are classified as Neanderthal, and the segments detected in both runs are classified as Denisovan. Since the second run integrates Eurasians into the outgroup, it effectively identifies archaic tracts that are in Papuans and absent in Eurasia, which are assumed to be from Denisovans. In a recent study, Yermakovich et al. (2024) also applied hmmix to Papuan genomes, and they ran hmmix once followed by additional filterings to distinguish between Neanderthal and Denisovan segments. This led to some unexpected results; despite Papuans harboring more Denisovan ancestry, the majority of archaic segments identified are genetically closer to the Neanderthal genome than the Denisovan genome.

Another difference between studies is whether Papuans have experienced two introgression events from distinct Denisovan populations. For example, Jacobs et al. (2019) found evidence for two introgression events, but Browning et al. (2018) does not identify evidence for more than one Denisovan introgression. There are several reasons why studies have different results. They may be due to differences in post-processing steps required to classify archaic segments into Denisovan or Neanderthal segments or the fact that Denisovan and Neanderthal ancestry are not jointly inferred. Here, we present a generalized model (DAIseg) that jointly detects multiple ancestries in a single analysis, which enables the simultaneous identification of Neanderthal and Denisovan ancestry. We also show how this model provides a framework to study archaic ancestry in recently admixed genomes.

In this study, we benchmark our method using simulated data to evaluate its power to detect introgression and compare it to hmmix. We also apply our model to Papuan genomes—known to harbor both Neanderthal and Denisovan ancestry—and demonstrate that it can infer these ancestries simultaneously. Moreover, our method identifies additional introgressed segments not identified by hmmix. Our analysis of introgressed segments in Papuan genomes supports the hypothesis of two distinct Denisovan introgression events, consistent with previous findings. Additionally, the inferred Denisovan segments show greater genetic similarity to the sequenced Denisovan genome, as expected. Notably, these results are achieved through a single application of our method, reducing the need for the complex filtering and post-processing steps required by earlier approaches.

In summary, our approach offers a streamlined and powerful framework for jointly detecting and classifying archaic genomic segments as Denisovan or Neanderthal in origin. Applying this method to Papuan genomes, we identified more archaic fragments and uncovered clear evidence of two separate Denisovan introgression events. Finally, we show how our framework can be extended to deconvolute both archaic and recent modern human ancestry within recently admixed individuals. Our results highlight the utility of our method for advancing our understanding of the role of archaic ancestry in modern human evolution.

## Model

### General overview

In this subsection we provide intuition for the key ideas underlying the algorithm and in the next subsection “HMM architecture” we describe the mathematical details. Our method generalizes hmmix (Skov et al. 2018) to enable the inference of archaic ancestry from multiple archaic populations. The main idea underlying hmmix is that archaic genomic segments introgressed into a population are expected to have a high density of private variants relative to modern human populations which did not interbreed with Neanderthals or Denisovans. In hmmix, the genome is binned into windows of fixed length. Each window is considered to be in one of the two hidden states, ingroup and archaic, representing anatomically modern human, Neanderthal, or Denisovan local ancestries, respectively. To construct observations, the number of private variants (genetic variants present in the haplotype of interest but absent in all of the reference genomes) is counted in each window. Emission probabilities are modeled using the Poisson distribution parameterized by the mutation rate and the mean coalescent time between ancestries. Transitions between hidden states are caused by recombinations, followed by ancestral lineages possibly switching their ancestry, depending on the timing and proportion of admixture.

Our method generalizes hmmix by allowing complex population scenarios with multiple reference and ancestral populations as well as multiple admixture events. The target population is the population in which we are detecting introgressed/admixed segments for some of its individuals (similar to an ingroup in hmmix). Reference populations are populations used to construct observations by identifying sets of private variants of a target genome relative to each of them (similar to an outgroup in hmmix). Ancestral populations define the set of hidden states, and they correspond to the source ancestries that one wishes to identify. Reference populations can be either non-admixed outgroups as in hmmix as well as genomes representing ancestral populations (the benefit of including even a small number of archaic genomes in the analysis is shown in Planche et al. 2024).

A population discriminates well between two ancestries if its average coalescence times with the two ancestral populations (taken separately) differ substantially. Our model distinguishes between *N* different ancestries using *M* reference populations. In theory, there is no necessary relationship between *N* and *M*. For example, it is possible to distinguish between more than two ancestries using a single reference population, or to use multiple reference populations to distinguish between only two ancestries. In practice, when building the model, the reference populations should be chosen such that each discriminates well between at least two ancestries.

Let us now give two toy examples to illustrate the model. Demography 1, illustrated in Fig. 1, approximates the Papuan population, which has genomic segments from both Neanderthals and Denisovans. Assume that we want to discriminate between non-archaic, Neanderthal and Denisovan ancestries in the target Papuan population. We use modern Africans and modern Eurasians as reference populations. We partition the genome into small windows (in practice of size 1 kb), and for each window, we count two numbers. The first one is the number of variants present in the target individual and absent in Africans. The second one is the number of variants present in the target individual and absent in Eurasians. Very roughly and intuitively, here is how the three ancestries will be detected. Non-archaic segments are expected to have few variants absent in Africa, and even fewer variants absent in Eurasia. This is because Papuans separated from Africa at an older time than they separated from other Eurasian populations. Neanderthal segments are expected to have a large number of variants absent in Africa, because archaic split time is much older than out of Africa split. The number of variants absent in Eurasia is still relatively small, because Neanderthals also introgressed into Eurasians. For a segment of Denisovan ancestry, we expect a large number of variants that are absent both in Africa and in Eurasia. Notice that we still expect fewer private variants relative to Eurasians than relative to Africans, because the former have Neanderthal component which shares an ancestral population with Denisovans.

**Figure 1:**
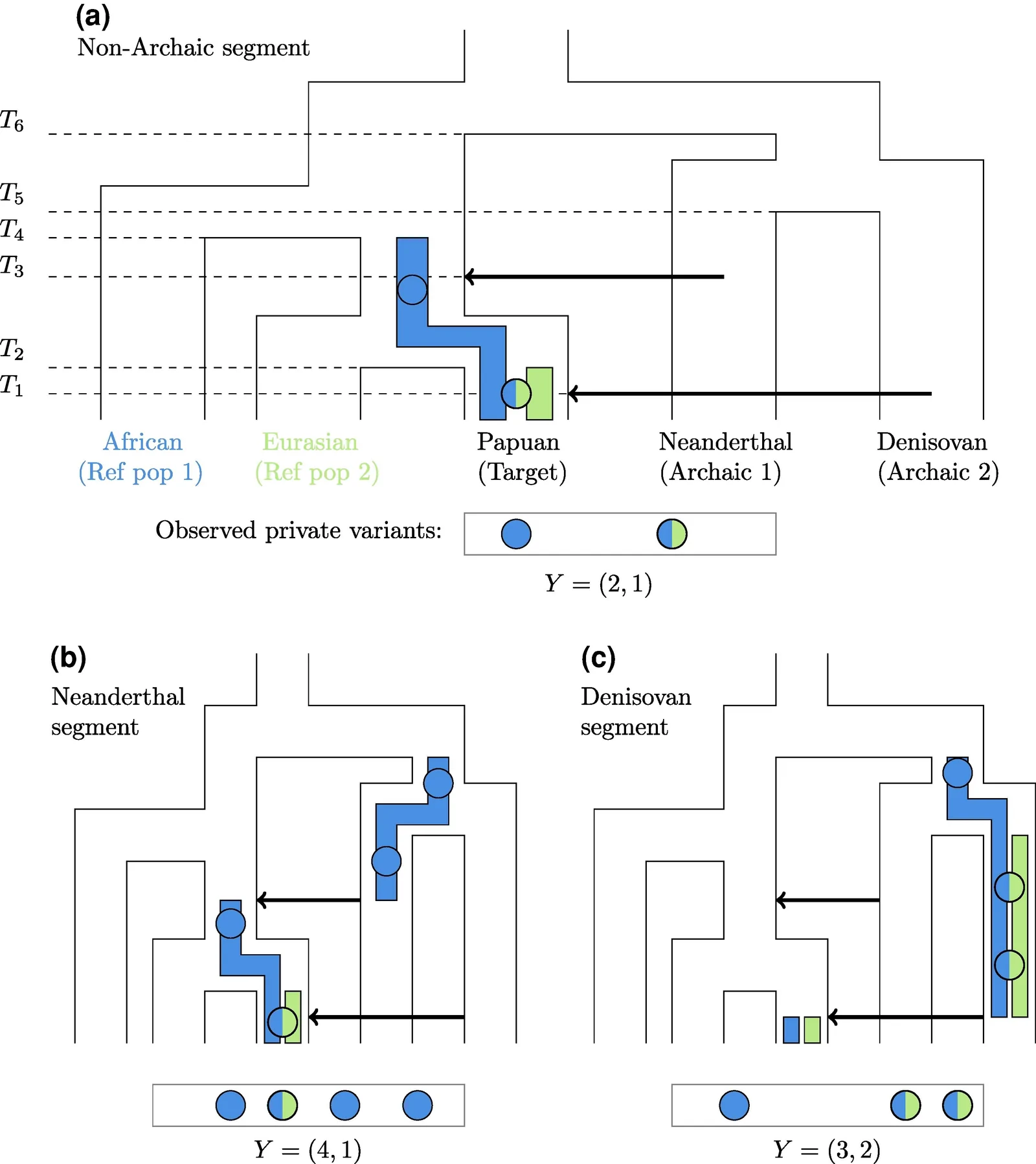
Demography 1: Two introgression events into modern humans. First, there is gene flow from Neanderthals into a non-African population which then split into Eurasian and Papuan. Second, Denisovans introgressed only into Papuans. The proportion of Neanderthal and Denisovan ancestry are, respectively, 3% and 4%. The HMM infers between three possible ancestries: Papuan, Neanderthal, or Denisovan. No reference genome from the archaic populations is used, but only from African and Eurasian. The times, in generations, are $T_{6}=30,000$, $T_{5}=24,000$, $T_{4}=2,400$, $T_{3}=2,000$, $T_{2}=1,700$, and $T_{1}=1,500$. The three demographic histories represent the histories of different segments, a) a non-introgressed segment, b) a Neanderthal introgressed segment, and c) a Denisovan introgressed segment. The blue and green lines show when the target lineage is completely separated from the corresponding blue/green reference population. Circles represent mutations that accumulate along each lineage: blue circles indicate mutations private with respect to the African populations, and half-green half-blue circles indicate mutations private to both African and Eurasian populations. $Y=(y_{1},y_{2})$ is a vector where $Y_{1}$ is the observed number of mutations absent in African and present in the target population (Papuans) and $Y_{2}$ is the observed number of mutations absent in Eurasia, and present in the target population. The exact number of private variants with respect to each ancestry is purely illustrative here.

With the second example, demography 2 illustrated in Fig. 2, we illustrate how the model can jointly detect archaic introgression and modern admixture. Assume a target population such as admixed Americans, which has ancestries from Africans and Indigenous Americans who harbor Neanderthal genomic segments. Assume we are inferring African, Neanderthal and Indigenous segments in that population. We once again use modern Africans and modern Eurasians as reference populations. For each genomic window, we count two numbers: the number of variants present in the target individual and absent in Africans and the number of variants present in the target individual and absent in Indigenous Americans. Intuitively, an unadmixed (Indigenous) segment is expected to have a few private variants relative to Eurasians and a larger number of variants relative to Africans. Segments of admixed (African) ancestry are expected to have very few variants that are absent in Africa, but more variants that are absent in Eurasia. Within archaic (Neanderthal) segments, there are a large amount of variants absent in Africa, but much fewer absent in Eurasian.

**Figure 2:**
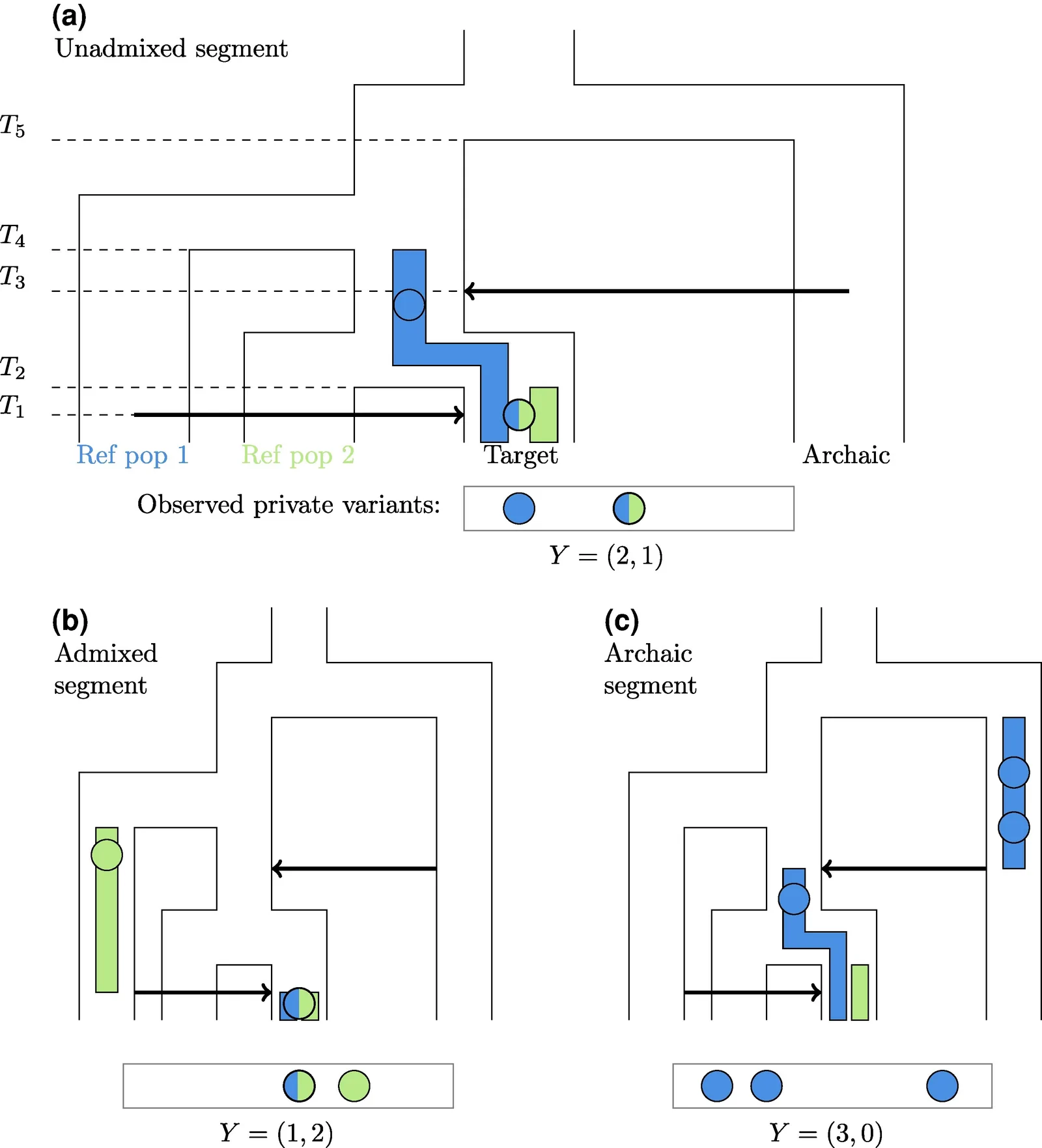
Demography 2: One archaic population introgresses into a modern human population which then splits into the Reference population 2 and target. The reference population 1 receives no archaic introgression, but later (more recently) admixes into the target population. The HMM infers three possible ancestries : reference pop 1, reference population 2, or archaic. The times, in generations, are $T_{5}=30,000$, $T_{4}=2,400$, $T_{3}=2,000$, $T_{2}=1,200$, and $T_{1}=40$. The three demographic histories represent the histories of different segments, a) an unadmixed segment, b) an admixed segment, and c) an archaic introgressed segment. The blue and green lines show when the target lineage is completely separated from the corresponding blue/green reference population. Circles represent mutations that accumulate along each lineage: blue circles indicate mutations private with respect to the first reference populations, green circles indicate mutations private to the second reference population and half-green half-blue circles indicate mutations private to reference populations. $Y=(y_{1},y_{2})$ is a vector where $Y_{1}$ is the observed number of mutations absent in the first reference population and present in the target population and $Y_{2}$ is the observed number of mutations absent in the second reference population, and present in the target population. The exact number of private variants with respect to each ancestry is purely illustrative here.

These two examples will be used to demonstrate our method performance based on simulations in section “Simulations.”

### HMM architecture

We infer the ancestry of a target haplotype along the genome based on an HMM. The haplotype is partitioned into *W* consecutive windows of fixed size *L* (in practice L=1 kb), indexed by w=1,…,W. The model is fully specified by a state space, an observation space, an initial distribution, transition probabilities, and emission probabilities, defined below; the derivations of the transition and emission probabilities are given in sections “Emission probabilities” and “Transition probabilities.”

**Notation.** Let *μ* and *r* be the per-base-pair, per-generation mutation and recombination rates, respectively. The model uses *M* reference populations $o_{1},\ldots ,o_{M}$ to distinguish between *N* ancestries. For an ancestry *x* and reference population $o_{i}$, let $T_{x,i}^{c}$ denote the genome-wide average of the coalescence time between a lineage of ancestry *x* and the most closely related lineage in $o_{i}$, that is, the minimum coalescence time over all haplotypes sampled from $o_{i}$.

Time is measured backward from present (t=0) into past (t>0) following coalescent tradition. Thus a time *t* is “more recent” than $t^{\prime }$ when $t<t^{\prime }$.

For each ancestry *x*, let $a_{x}$ be its admixture proportion, the proportion of recipient population substituted by individuals from the admixed (introgressed) population; by convention the target’s own ancestry has $a_{x}=1$. Let $a_{x}(t)$ denote the fraction of ancestry *x* in the target population at time *t* (section “Transition probabilities”).

**State space.** Let $S=\{x_{1},\ldots ,x_{N}\}$ be the state space of the HMM. The hidden state at window *w* is the ancestry $q_{w}\in S$ of that window. Each ancestry corresponds to one possible path of the ancestral lineage of the target haplotype, from the present back to the root of the population scenario.

**Observation space.** For each window *w* and for each reference population we count the number of private derived variants carried by the target haplotype and absent from that reference population. This gives the observation $Y_{w}=(y_{w,1},\ldots ,y_{w,M})\in N^{M}$ for the window *w*, where $y_{w,i}$ is the count relative to population $o_{i}$. Intuitively, a large value of $y_{w,i}$ reflects a deep coalescence time between the window’s ancestral lineage and population $o_{i}$, which is the signal the emissions exploit.

**Initial distribution.** The initial state follows the genome-wide ancestry fractions: $\pi_{x}=P(q_{1}=x)=a_{x}(0)$, the present-day fraction of ancestry *x* in the target population, with $\sum_{x\in S}a_{x}(0)=1$.

**Emission probabilities.** For all *w*, $b_{x}(Y)=P(Y_{w}=Y|q_{w}=x)$ is the probability of the private-variant tuple *Y* given ancestry *x* at window *w*. We present two approaches to construct these emission probabilities. The first one approximates the *M* entries of the tuple *Y* as independent random variables, which already provides good accuracy and is derived in section “Emission probabilities.” In supplementary material S1, we present the second approach which takes into account the dependency under certain scenarios and highlights its slight improvement. Our real data analysis relies on this more accurate model.

**Transition probabilities.**  $P(x\to x^{\prime })=P(q_{w+1}=x^{\prime }|q_{w}=x)$ is the probability that two adjacent windows switch ancestry from *x* to $x^{\prime }$. It is driven by recombination and depends on *L*, *r*, the admixture times and the ancestry fractions $a_{x}(t)$. The probabilities are derived in Section “Transition probabilities.”

**Parameter estimation.** We derive the EM algorithm to estimate parameters of the HMM. Notice that the well-known Baum–Welch algorithm cannot be used here because of the special parametrization based on the population history. Details are given in supplementary material S4.

**Decoding and segment calling** Once the model parameters are set, we assign an ancestry to each window using posterior decoding obtained by the forward–backward algorithm. A window is called ancestry *x* when the posterior probability exceeds a fixed threshold. Windows in which no ancestry reaches the threshold are left with the target ancestry (no admixture/introgression). Adjacent windows sharing the same called ancestry are merged into admixed/introgressed segments. Raising the threshold makes calls more conservative, trading recall for precision for admixed/introgressed ancestries. Unless stated otherwise, we report results at threshold of 0.5. To illustrate the trade-off between precision and recall we also report results for threshold of 0.98 for simulations under demography 1 (Table 1).

**Table 1:** Comparison of DAIseg and hmmix methods for posterior detection of two components of archaic introgression (neanderthal and denisovan) based on 40 simulated haploid individuals (demography 1).

		Posterior probability threshold					
		0.5			0.98		
		Precision	Recall	F-score	Precision	Recall	F-score
DAIseg	Neanderthal	0.92	0.75	0.83	0.93	0.74	0.82
	Denisovan	0.82	0.96	0.88	0.83	0.95	0.89
	Archaic	0.96	0.996	0.98	0.98	0.99	0.98
hmmix	Neanderthal	0.94	0.62	0.75	0.96	0.64	0.77
	Denisovan	0.75	0.99	0.85	0.78	0.98	0.87
	Archaic	0.97	0.996	0.98	0.99	0.98	0.98

Precision, recall, and F-score for Neanderthal, Denisovan, and archaic segments are given for each method with posterior threshold values of 0.5 and 0.98. Archaic segments are those classified as either Neanderthal or Denisovan.

#### Emission probabilities

Our first approximation of emission probabilities assumes that all entries of the observation tuple are independent. Hence, for an ancestry *x*, the probability of observing $Y=(y_{1},y_{2},\ldots ,y_{M})$ is computed by multiplying the probabilities of observing each $y_{i}$ given that the ancestry of the window is *x*. Under an infinite site model, the number $y_{i}$ of private variants relative to the population $o_{i}$ follows the Poisson distribution with the parameter $\lambda_{x,i}=\mu \cdot L\cdot T_{x,i}^{c}$. Let $Z_{x,i}$ be a discrete random variable following a Poisson distribution with this parameter $\lambda_{x,i}$. Then emission probabilities are


$$b_{x}(Y)=P(Y|x)=\prod_{i=1}^{M}P(Z_{x,i}=y_{i}).$$

In supplementary material S1, we detail how to take into account the dependency between the tuple entries $y_{i}$.

#### Transition probabilities

For the transition probabilities, we expand the ideas from Skov et al. (2018) to scenarios with multiple admixture events. We assume that recombinations might happen between two adjacent windows but not within a window. The transition probabilities depend on the window size *L*, recombination rate *r*, timings of admixtures, and on the fractions of each ancestry in the genome. In Skov et al. (2018), the demographic model has a single admixture event, and two hidden states (archaic and non-archaic ancestry), so the fraction of archaic ancestry is constant over time and equal to its estimated introgression proportion. There is an important difference in our model, because we consider multiple admixture events (eg Figs. 1 and 2). Each new admixture event (here discussed forward in time) dilutes the ancestries which are already present in the recipient population. This makes our transition probabilities different from Skov et al. (2018). The following paragraph explains how to compute each ancestry fraction through time, using demographic parameters, and finally derive transition probabilities.

**Dilution of ancestry fractions.** Let $T_{0}^{a}=0$ and denote $T_{1}^{a}<T_{2}^{a}<\cdots <T_{N-1}^{a}$ the admixture times. Introduce the notation $\tau_{x}$ which maps any ancestry *x* to its admixture time $T_{j}^{a}$ for some j∈{1,…,N−1}. Also by convention assume that for the target population ancestry $\tau_{x}=\infty$. Let $a_{j}$ be the admixture proportion associated with the admixture event at time $T_{j}^{a}$. When a population receives successive pulses of admixture, each new pulse dilutes the ancestries already present. Suppose ancestry *x* entered the target at time $\tau_{x}$ with proportion $a_{x}$. If it follows by another admixture event (more recent than $\tau_{x}$) which introduces a fraction $a_{j}$ of some other ancestry, then a fraction $a_{j}$ of the recipient population is replaced, and only $\left(1-a_{j}\right)$ of it retains its previous composition. The fraction of *x* is therefore scaled by $\left(1-a_{j}\right)$. Each admixture event more recent than $\tau_{x}$ applies such a factor independently, so the fraction of ancestry *x* at time *t* is its initial proportion multiplied by one $\left(1-a_{j}\right)$ factor per intervening event. Therefore for any time *t* more recent than $\tau_{x}$,


$$a_{x}(t)=a_{x}\prod_{\;j\;:\;t<T_{j}^{a}<\tau_{x}}(1-a_{j}).$$

This formula does not take into account genetic drift, so in fact $a_{x}(t)$ represents the expectation of the real admixture fraction over time.

**Derivation of transition probabilities.** Now that we have each ancestry fraction in the target population through time, we can compute the probabilities of recombination events in a similar fashion as in Skov et al. (2018). We begin with splitting time into intervals $\left[T_{j}^{a},T_{\;j+1}^{a}\right]$ and compute the probability of a recombination event happening in each of these intervals.

With the recombination rate *r*, for a given window, the number of recombination events during time *T* is Poisson distributed with rate r⋅L⋅T. Therefore, the probability of having at least one recombination is $1-e^{-r\cdot L\cdot T}$ which can be approximated by r⋅L⋅T. Let *x*, $x^{\prime }$ be two ancestries. Any recombination event switching between these two ancestries can only happen for times more recent than $T_{x,x^{\prime }}^{a}=\min(\tau_{x},\tau_{x^{\prime }})$. The recombination event which switches ancestry from *x* to $x^{\prime }$ should be in a time interval $\left[T_{j}^{a},T_{\;j+1}^{a}\right]$, with $T_{\;j+1}^{a}\leq T_{x,x^{\prime }}^{a}$. The probability that the new ancestry is $x^{\prime }$ approximately equals to the ancestry fraction $a_{x^{\prime }}(T_{j}^{a})$ within this interval. Finally, it is required that there is no more recent recombination within the interval $\left[0,T_{j}^{a}\right]$ which is equal to $e^{-r\cdot L\cdot T_{j}^{a}}\approx 1-r\cdot L\cdot T_{j}^{a}$. Finally,


$$P(x\to x^{\prime })=\sum_{\;j\;:\;T_{\;j+1}^{a}\leq \min(\tau_{x},\tau_{x^{\prime }})}\left(r\cdot L\cdot (T_{\;j+1}^{a}-T_{\;j}^{a})\cdot a_{x^{\prime }}(T_{j}^{a})\right)(1-r\cdot L\cdot T_{j}^{a}).$$

The probability of remaining in the same ancestry is


$$P(x\to x)=1-\sum_{x^{\prime }\in S\setminus \{x\}}P(x\to x^{\prime }).$$

## Results

### Simulations

We illustrate the flexibility and performance of our method by running simulations, using msprime (Kelleher et al. 2016), under two demographic scenarios shown in Figs. 1 and 2. The first scenario models two pulses of archaic introgression from two different archaic populations. The second scenario models one archaic and one modern human admixture. Moreover, for the scenario 2 reference population 2 is both an ancestry and a reference population. The script details and availability can be found in supplementary material S2.

#### Demography 1

We compute the accuracy of our method on the first demographic model (Fig. 1), corresponding to the case where a Papuan population has both Neanderthal and Denisovan ancestry in significant proportions (respectively, 3% and 4% Reich et al. 2010; Witt et al. 2023). We wish to distinguish between three different ancestries, Neanderthal, Denisovan, and AMH. To do so, as in Skov et al. (2018), we will use two outgroups, Africans (reference population 1) which received no archaic introgression and Europeans+East Asians (Reference population 2) which received introgression from Neanderthal (archaic 1), but no or very little from Denisovan (archaic 2). The African population allows us to discriminate AMH ancestry from archaic while the Eurasian population allows us to discriminate Denisovan ancestry from the rest. We simulate a 250 Mbp chromosome for 150 diploid individuals for each reference population and 20 diploid individuals for the target population and compare the performance of hmmix (Skov et al. 2018) and our model, DAIseg, on this dataset. We used a split time between archaic and modern populations at 30,000 generations ago, a split time between Denisovan and Neanderthal at 24,000, out of Africa expansion at 2,400, the admixture time of Neanderthal at 2,000 (Prüfer et al. 2013), a split time between Papuans and Eurasians at 1,700 (Malaspinas et al. 2016), and Denisova admixture time at 1,500 (Sankararaman et al. 2016).

For this demography, both in simulations and in the real data (section “Application to Papuan genome”) we use DAIseg with emission probabilities as defined in supplementary material S1. As hmmix cannot distinguish between more than two ancestries, we run it twice, first to detect archaic segments, using African population as outgroup and second to detect Denisovan segments, using the union of African and Eurasian populations as outgroup. Afterwards, the segments that were classified as archaic, but not Denisovan are classified as Neanderthal. The results (precision, recall, and F-score) are shown in Table 1. Precision is the ratio of correctly predicted positive observations over the total predicted positives, ie $\frac{TruePositive}{TruePositive+FalsePositive}$, Recall is the ratio of correctly predicted positive observations over all the observations in the actual class, ie $\frac{TruePositive}{TruePositive+FalseNegative}$ and the F-score is a summary statistic of precision and recall defined as $2\cdot \frac{Precision\cdot Recall}{Precision+Recall}$.

We show that combining all the information about outgroups and reference populations in a single model avoids extra post-processing steps while slightly increasing the accuracy of the inference, as measured by the F-score. Note that when increasing the posterior probability cut-off of the HMMs from 0.5 to 0.98, which is the minimal outputted probability above which we assign an introgressed ancestry, we expect an increase in Precision, but a decrease in Recall. This is true for all cases but one, detecting Neanderthal segments using hmmix, where both precision and recall increase with the cut-off. Understanding this phenomenon will help intuit the advantages of combining all reference populations in a single HMM. Indeed, the most common reason why Neanderthal segments are underdetected in this particular demographic scenario is that they are mistaken for Denisovans (see confusion matrices Fig. S2). By increasing the cut-off in hmmix, one Neanderthal segment which was misclassified as Denisovan, might not be anymore, but still be detected as archaic. In other words this segment looked like Denisovan, but even more so Neanderthal. While our model will use this combined information to classify it as Neanderthal, two independent runs of hmmix will simply classify it as both archaic and Denisovan, hence Denisovan.

**Unphased data**

The method assumes that the data is phased, but we show with simulated data that it can be used on unphased data as well. When constructing the observations, we simply count all the private variants in the window. Under the current demography, the model still outputs three possible ancestries, modern, Neanderthal, or Denisovan while in fact any position in the genome has two ancestries (from each haplotype). In particular, if a position has both Denisovan and Neanderthal ancestries, the model will not be able to detect both ancestries and the recall score will inevitably decrease. It will still very likely be detected as Denisovan or Neanderthal, hence correctly identified as archaic. We ran the model on simulated unphased data. We consider the true ancestry to be archaic if the position has at least one archaic haplotype. If an individual harbors both Neanderthal and Denisovan ancestries at a specific position, we do consider that the position has both, which means that the model can correctly call it archaic (by predicting either Neanderthal or Denisovan), but will necessarily fail to detect one of the two ancestries. The results are shown in Table 2, using unphased data mostly decreases the precision score for the archaic ancestries. This result can be intuitively understood. When using unphased data, we are summing the private variants present on both copies of the chromosome, increasing the number of observations per window. On this particular demographic model, a large number of observations per window is a sign of archaic ancestry.

**Table 2:** Precision and recall scores for DAIseg applied to phased and unphased simulated data.

	Phased			Unphased		
	Precision	Recall	F-score	Precision	Recall	F-score
Neanderthal	0.92	0.75	0.83	0.91	0.73	0.81
Denisovan	0.82	0.96	0.88	0.77	0.95	0.85
Archaic	0.96	0.996	0.98	0.94	0.997	0.97

Twenty Papuan-like diploid individuals were simulated using msprime (Kelleher et al. 2016) with two introgressed archaic populations corresponding to Neanderthals and Denisovans. We use posterior decoding with a 0.5 cutoff.

**Estimation of demographic parameters**

For parameter initialization, the model uses estimated admixture times and split times as approximations of coalescent times to compute the emission probabilities. While in simulations these parameters are known, in reality, they can only be first estimated, be it by other genomic analyses or archeological findings.

The most common way to estimate HMM parameters is with the Baum–Welch algorithm which is known to be very sensitive to initialization of parameters, quickly falling in local extrema (Durbin et al. 1998). The Baum–Welch algorithm is an expectation-maximization (EM) algorithm which assumes that the model parameters are independent and infers them as such. In our case, the emissions parameters are not independent and depend on a few demographic parameters. In the present demography, these parameters are: split time between archaics and moderns, split time between Neanderthals and Denisovas, split time between Eurasians and Africans (Out Of Africa) and split time between Eurasians and Papuans. We developed an EM-algorithm that co-estimates the emission parameters by jointly inferring these demographic parameters. It shows very good estimates even when the initial estimate of the parameters were off by up to 80%. The full mathematical derivations are given in supplementary material S4 and the simulation results are shown in Fig. S1. This allows to keep very good inference accuracy even when the demographic parameters are largely (up to 80%) miss-estimated (Table S2). We also show that even without parameter estimation, the model is very robust to misestimation of the parameters, with close to no loss of performance (as measured with F-score) when parameters are misestimated within a ±50% range and only a maximum decrease of the F-score of 0.06 when the parameters can be off by up to ±80% (Table S2).

#### Demography 2

We now test the method on the second demographic model. In this case the target population received both an archaic admixture and a modern human one, with admixture proportions, respectively, 3% and 15%. The archaic population split from modern humans 30,000 generations ago and admixed 2,000 generations ago. The reference population 1 split from the target population 2,400 generations ago and admixed 40 generations ago. In Fig. 2, we propose two reference populations to distinguish between the three possible ancestries in the target population. One can note that reference population 1 already theoretically contains enough information to infer between the three ancestries. But due to the stochastic nature of the process, which can, for example, lead to incomplete lineage sorting, it is better to use as much relevant data as possible. Using reference population 2 offers extra information which reduces uncertainty. This illustrates our previous point when presenting the HMM model, there is no direct relationship between the number of reference populations and the number of ancestries, our model can take into account any relevant information available. To illustrate this, we first run DAIseg using only Reference population 1 (the observations are then one integer per window) and then using both Reference populations 1 and 2 (the observations are then two integers per window), all other parameters being equal. The results are summarized in Table 3. Notice that, using only one reference population our method already achieves high accuracy for distinguishing between three ancestries. Adding a second reference population does indeed increase the overall inference accuracy. At the same time this additional reference population 2 does not help to increase the accuracy for the detection of archaic segments (precision of 0.94 and recall of 0.97 in both cases). The reason being that reference population 1 already has a very large divergence time with that population, yielding a high number of private variants. On the other hand, using reference population 2 helps to distinguish between reference pop 2 and reference population 1 segments.

**Table 3:** Precision and recall scores for DAIseg applied to the simulated data under demography 2.

	Reference populations used					
	Reference pop 1			Reference pop 1 and reference pop 2		
	Precision	Recall	F-score	Precision	Recall	F-score
Reference pop 2	0.88	0.99	0.93	0.97	0.99	0.98
Archaic	0.94	0.97	0.95	0.94	0.97	0.95
Reference pop 1	0.997	0.8	0.89	0.998	0.95	0.97

In the left column only reference population 1 was used to construct observations. In the right column reference populations 1 and 2 were jointly used for the inference which helps to better distinguish between reference pop 2 and reference population 1 ancestry. We use posterior probability with cut-off 0.5.

### Application to Papuan genome

We apply our model to 16 Papuan individuals—who harbor Denisovan and Neanderthal ancestry—from the Simons Genome Diversity Project (Mallick et al. 2016) to detect and classify introgressed segments as being of Denisovan or Neanderthal origin in real genomic data. We use two reference populations, the first consists of 205 Africans from 1,000 Genomes Project (Auton et al. 2015), from Yoruban (YRI) and Esan (ESN) populations. The second reference population consists of 814 Eurasians from British (GBR), Finnish (FIN), Toscan (TSI), Utah (CEPH) with Northern and Western European ancestry populations, Japanese (JPT), and Han Chinese (CHB). Our HMM has three hidden states, Target (Modern), Neanderthal and Denisovan. Our underlying assumptions in choosing our outgroup populations are that African populations do not carry Neanderthal or Denisovan ancestry and that African+Eurasian populations do not harbor Denisovan ancestry and share a single Neanderthal introgression event. For segment inference, we use posterior decoding with a cutoff of 0.5.

We compare our model to hmmix, which was ran twice; the first time using Africans as an outgroup, and the second time using Africans and Eurasians as the outgroup. We followed the procedure of Skov et al. (2018) for classifying archaic segments into Denisovan or Neanderthal: archaic segments detected only in the first run are classified as Neanderthal and archaic segments detected in both runs are classified as Denisovan. Using DAIseg, we detect 24% more Neanderthal segments and 17% more Denisovan segments than hmmix. Out of all Neanderthal segments detected with hmmix, we classify 82% of them as Neanderthal, 5% as Denisovan, and 13% as non-archaic. For Denisovan segments detected with hmmix, we classify 93% of them as Denisovan, 1% as Neanderthal and 6% as non-archaic. To check if those differences are the result of misclassification, we compare the similarity to archaic reference genomes of segments detected only by DAIseg, only by hmmix or by both methods. Here, for each segment, the similarity is computed by counting the number of single-nucleotide polymorphisms (SNPs) in the segment where an allele is shared between the target Papuan and the archaic (either Vindija Neanderthal or Altai Denisovan), then dividing by the total number of SNPs present in the segment (supplementary material S5). Overall, our model detects more archaic segments and closer inspection (Fig. 3) shows that these are mostly of archaic origin and not the result of misclassification. Figure 3 also shows that Neanderthal segments detected with DAIseg have slightly higher genetic similarity to the Neanderthal genome than Neanderthal segments detected with hmmix. For Denisovan segments identified with DAIseg, genetic similarity to the Denisovan genome is slightly lower than for Denisovan segments detected with hmmix. We compare the length (Fig. S4) of the Neanderthal and Denisovan segments. We find the Neanderthal segments to be slightly longer than the length of detected Denisovan segments. The introgression maps for Papuans are available in supplementary material S6.2.

**Figure 3:**
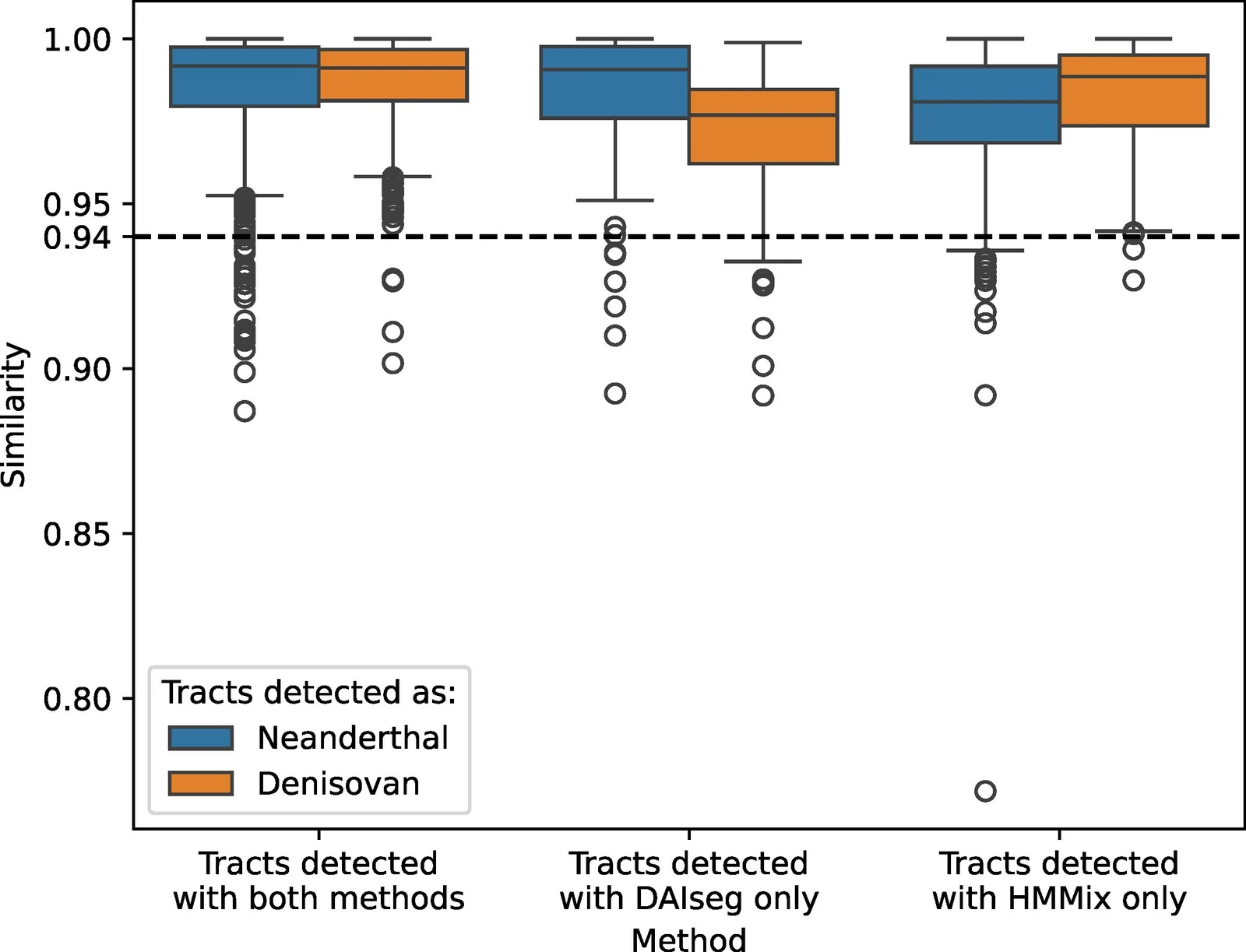
Similarity of the segments detected as Neanderthal (resp. Denisovan) to the Vindija Neanderthal (resp. Altai Denisovan) reference genome. On the left, the similarity computed for segments that are detected with both DAIseg and hmmix, in the middle the segments that are only detected with DAIseg, on the right, the segments only detected with hmmix. Segments detected with both methods account for 66% of all segments, segments detected only with DAIseg for 26% and detected only with hmmix for 8%. Here, all SNPs present in the segments are used to compute the similarity, non-archaic segments have a similarity of around 0.94 to archaic reference genomes.

As previous studies have identified evidence for two Denisovan introgression events into Papuans (Choin et al. 2021; Yermakovich et al. 2024), we analyzed Denisovan segments detected using DAIseg to determine if two subsets of Denisovan segments exhibit varying levels of genetic similarity to the sequenced Denisovan genome. The rationale behind this analysis (proposed notably in Browning et al. 2018) is that if segments were introduced from a single introgression pulse, then they share a common source and admixture time, so the distribution of segment similarity to an archaic reference genome should have a single mode. For this analysis, we computed genetic similarity using archaic private variants (see supplementary material S5) to avoid using the same exact set of positions employed by the model, thereby reducing the risk of circular reasoning. We then used contour plots to visualize the relationship between the inferred introgressed segments and the sequenced Neanderthal and Denisovan genomes. This visualization also offers another way to check the accuracy of our model. In Fig. 4 and Fig. S3, we take Denisovan and Neanderthal segments detected with DAIseg and compute the genetic similarity to the Denisovan genome (*y* axis) and the Vindija Neanderthal genome (*x* axis). On average, we expect Denisovan segments to appear above the diagonal, which means more similarity to the sequenced Denisovan genome than to the sequenced Neanderthal genome, and this is what we observe. For Neanderthal segments, we expect them to appear below the diagonal (see second panel of Fig. 4). Following Jacobs et al. (2019) to determine if there is evidence for two waves of Denisovan introgression, we kept all the segments detected as archaic with length greater than 200 kb and conditioned on observing at least ten private variants in the archaic genome (with respect to African populations). For completeness, we also give the same figure without any length threshold on the segments (Fig. S3).

**Figure 4:**
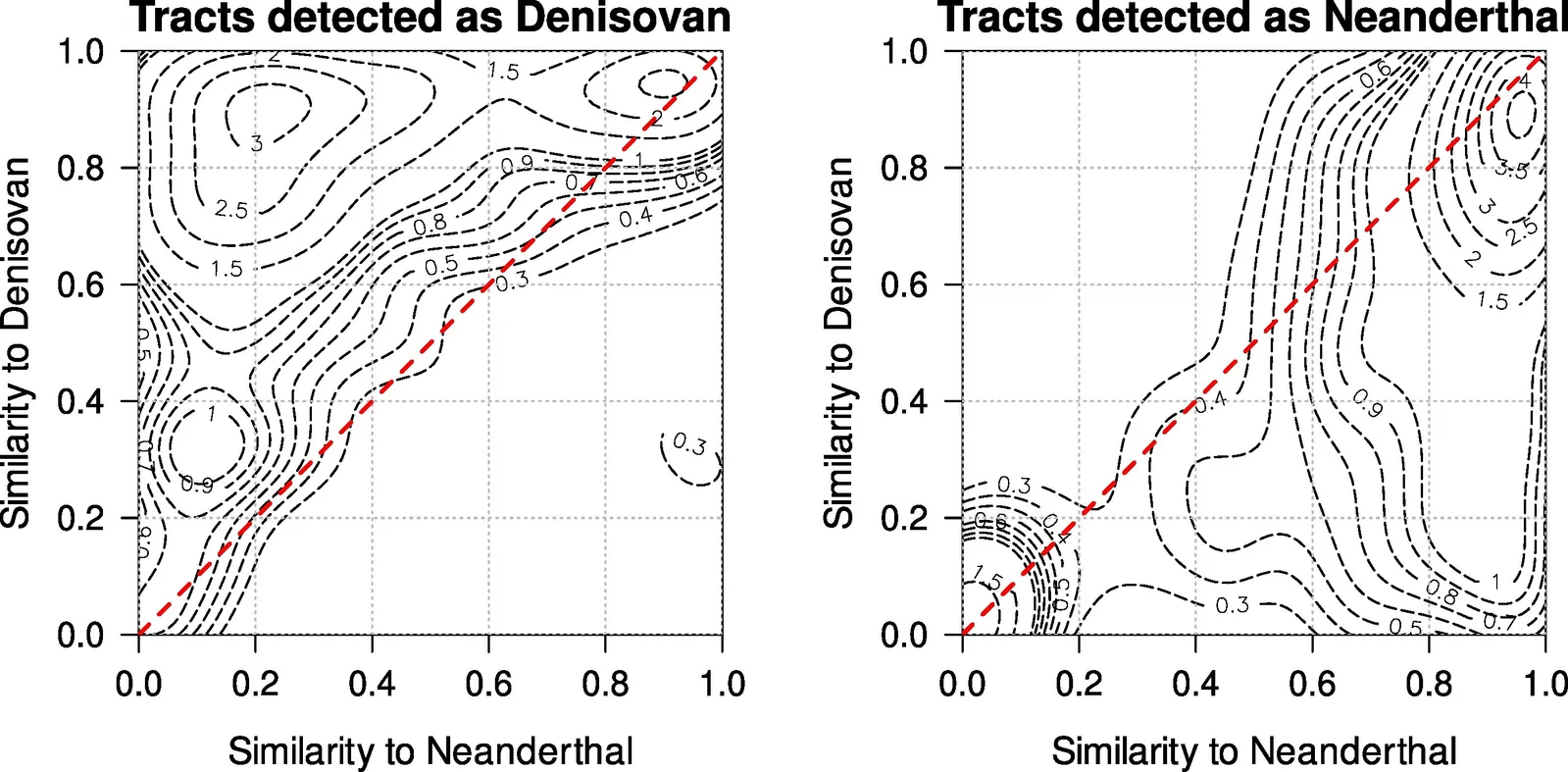
Measuring similarity to archaic genomes using the number of private variants in either Denisovan or Neanderthal. Segments of size larger than 200 kb, detected as Denisovan with DAIseg are shown in the left panel and those detected as Neanderthal in the right panel. The genetic similarity to the Vindija Neanderthal is shown on the *x* axis and to the Denisovan on the *y* axis. 15% of segments detected as Neanderthal and 6% of segments detected as Denisovan show a similarity lower than 0.2 to either archaic genome. The red dashed line, y=x, corresponds to an equal similarity to the Neanderthal and Denisovan reference genomes.

We observe that for both Neanderthals and Denisovan, there is a slight peak in the distribution around the origin, which means there is a set of inferred introgressed segments with low similarity to both of the archaic genomes. By defining that peak as having similarity below 0.2 to both archaic genomes, for all segments detected as Neanderthal, around 15% are at this peak, and for all segments detected as Denisovan around 6%. While we do not know the ground truth, these numbers can be interpreted as a rough estimate of the false discovery rate. In fact, when we look at segments detected as non-archaic, 97% have similarity below 0.2 to both archaic genomes. If we consider only smaller segments with length below 50 kb, the pseudo rate of false discovery is higher, but even then, it does not exceed 18% for Denisovan segments and 24% for Neanderthal segments (Fig. S3).

Apart from the peak around the origin, for tracts detected as Denisovan, we observe three peaks, one near (0.9, 0.9), one around (0.2, 0.9), and one around (0.1, 0.3), with the first number in parenthesis being the similarity to Vindija Neanderthal and the second, the similarity to Altai Denisovan. For tracts detected as Neanderthal, no such behavior is observed, the similarity to Neanderthal shows only one peak, at around 0.9. These observed varying levels of genetic affinity between inferred introgressed segments and sequenced archaic genomes are consistent with more than one introgression event with Denisovans and one introgression event with Neanderthals.

In the contour plot for Papuans in Browning et al. (2018), there is only one major peak for Denisovans and one for Neanderthals while our contour plot has multiple peaks. In particular, our contour plots have a peak around (0.9, 0.9) which suggests that there are some segments that are detected as Denisovan that are equally distant from Neanderthals and Denisovans. We note that our contour plots are not directly comparable to those presented in Browning et al. (2018) because both the inferred introgressed segments and the similarity metric differ between the two studies. We applied a similarity metric close to the one used in Browning et al. (2018) (supplementary material S5), and this results in 15% of segments detected as Denisovan being more similar to the sequenced Neanderthal (see Fig. S5) compared to the previous similarity measure. This shows that even after applying the metric close to the one used in Browning et al. (2018), we still observe some segments detected as Denisovan with high similarity to both archaic genomes. Finally, to compare as closely as possible with the contour plots of Browning et al. (2018), we restricted to regions supported by both methods. We intersected the DAIseg Denisovan segments longer than 80 kb with the archaic segments reported by Browning et al. (2018), retaining the intersected intervals. Match rates to the Denisovan and Neanderthal reference genomes were then computed on these intersections. Fig. S6 shows the similarity to archaic genomes using the private positions and match/mismatch information directly given in the Browning et al. (2018) maps (SPrime output). We notice that this contour plot resembles the one in Browning et al. (2018), and in this plot, the signal for two waves of introgression disappears. This suggests that the presence/absence of this signal is probably affected by the similarity measure used, rather than the introgression detection method. Notably, the similarity of these regions lies much closer to the Denisovan than the Neanderthal, further confirming the ability of DAIseg to distinguish between Denisovan and Neanderthal segments.

## Discussion

Here we present a new method, DAIseg, to detect introgressed segments from multiple archaic sources. Our framework is general and can be extended to detect introgression from several sources, including non-archaic ones. In the case of Neanderthal and Denisovan introgression (see demographic model in Fig. 1), and using simulated data, we showed that our performance is similar to or better than state-of-the-art methods. For example, compared to hmmix, we get a similar F-score for Denisovan (0.88 vs 0.85) and archaic detection (0.98 vs 0.98), but our performance is higher for Neanderthal detection (0.83 vs 0.75). One big advantage of our method is that DAIseg allows for the detection and classification of multiple ancestries with a single run of the HMM. While we mostly focused on Denisovan and Neanderthal introgression, we also showed how one can detect archaic ancestry in recently admixed individuals (demographic model 2, Fig. 2). Using simulations, we showed that we can accurately detect African, European, and archaic ancestry, suggesting that this framework can be used to deconvolute both archaic and modern human ancestry in recently admixed individuals.

Our method not only detects archaic segments, but it also classifies segments as being from a Denisovan or Neanderthal source. This is particularly useful when the target individuals harbor ancestries from both of these archaic sources. Here, we applied DAIseg to genomic data from Papuans who are known to carry ancestry from both Denisovans and Neanderthals. We find that our method identifies around 18% more introgressed segments than hmmix, likely because we are using more information when we jointly infer tracts. We infer the majority of these additional segments to be of Neanderthal origin, which we further confirmed by looking at the similarity of these tracts to archaic reference genomes (Fig. 3).

We note that around 25% of all inferred archaic segments have a very high similarity to both Neanderthal and Denisovan reference genomes. These segments are sometimes classified as Neanderthal and sometimes as Denisovan by our model (but always as archaic), showing that these may be shared segments or they might have a more complex demographic history. Importantly, the majority of archaic segments detected by DAIseg align with expected features. For example, Papuans harbor more Denisovan ancestry (3.6% of their genomes) than Neanderthal ancestry (2.8%), which is consistent with prior findings. Moreover, inferred Denisovan segments are genetically closer to the sequenced Denisovan genome than to the Altai Neanderthal genome, while inferred Neanderthal segments show greater similarity to the sequenced Neanderthal genome (Fig. 4). These observations confirm that DAIseg reliably classifies introgressed segments and provides a principled framework for segment detection without relying on extensive post-processing steps.

We note that a previous study (Yermakovich et al. 2024) presented unexpected results when detecting archaic ancestry in Papuans. They identified 60% of archaic segments in Papuans as being more similar to the Neanderthal genome, even though reported estimates of Denisovan ancestry in Papuans are higher than Neanderthal ancestry. However, many of the segments they detect exhibit only slightly lower similarity to the Denisovan genome, which may correspond to segments that we infer as Denisovan but also have a high genetic similarity to Neanderthals (top right peak in Fig. 4). By jointly inferring Neanderthal and Denisovan ancestry, DAIseg helps resolve such ambiguities and produces results that align with results in the literature, offering an improvement over prior approaches. Another example of this regards the number of Denisovan sources that contributed to ancestral Papuan populations.

In line with previous studies, our results provide clear evidence of two separate Denisovan introgression events. Specifically, when considering inferred Denisovan segments Fig. 4 reveals three peaks. One peak contains segments of high genetic similarity only to the Denisovan genome, another peak is made up of segments with high genetic similarity to both the Neanderthal and Denisovan genomes, and a third peak has intermediate genetic similarity to only the Denisovan genome. When considering segments detected as Neanderthal, we find that genetic similarity is closest to Neanderthal (see Fig. 4) although some segments also have high similarity to the Denisovan genome. These signatures suggest that at least three archaic contact events occurred in ancestral Papuan populations—two with Denisovans and one with Neanderthals. This additional Denisovan pulse was not detected in the contour visualization of the archaic segments inferred with Sprime in Papuans (Browning et al. 2018). This suggests that both the method used to infer introgressed segments and the approach used to measure genetic similarity to archaic genomes can influence the results. Finally, regarding the peak comprising segments that are highly similar to both Neanderthals and Denisovans, we currently interpret this as the result of having introgressed fragments that are present in both archaic populations due to shared ancestry or admixture events between archaic populations (Peter 2020). In Yermakovich et al. (2024) propose an alternative explanation for these archaic segments, where they suggest an introgression event from an archaic population that is closely related to both Denisovans and Neanderthals. However, as most analyses have harsh thresholds—often based on genetic similarity—these segments are often missed or excluded, and future analysis of all archaic genomes may reveal the history of these introgressed segments.

We acknowledge that our model requires carefully chosen outgroup populations and a well-defined demographic history. Although DAIseg does not explicitly use Denisovan or Neanderthal genomes for inference, the method can easily be extended to include them as reference populations, and it offers substantial flexibility to incorporate diverse demographic scenarios. For example, a recent study demonstrated that incorporating multiple Neanderthal genomes as references improves the detection of Neanderthal introgressed segments (Planche et al. 2024). While only a single Denisovan genome is currently available, the sequencing of additional Denisovan genomes in the future could further enhance the detection and classification of Denisovan ancestry. One exciting direction is to use DAIseg on ancient genomes (Capodiferro et al. 2025). This would require taking into account missing data, but as more ancient genomes are being imputed, using genotype probabilities might be sufficient. In sum, the success of our method in identifying multiple Denisovan introgression events in Papuans suggests its broader applicability. Applying DAIseg to other populations, such as South Asians and East Asians, where additional Denisovan introgression events have been proposed (Browning et al. 2018; Witt et al. 2022), may yield further insights into the complexity and history of archaic admixture.

## Supplementary Material

msag235_Supplementary_Data

## Data Availability

The method is implemented in Python and is available for use at: https://github.com/LeoPlanche/DAIseg. The inferred introgression map for archaic segments in Papuans is available at: https://github.com/LeoPlanche/DAIseg/blob/main/src/IntrogressionMap. The file will be gradually completed as more data becomes available. The samples and archaic segments described in this article will be kept as they are.
