# Supplementary material for: An Archaic Reference-Free Method to Jointly Infer Neanderthal and Denisovan Introgressed Segments in Modern Human Genomes
Source: Mol Biol Evol. 2026 Sep 17;43(11):msag235. doi: 10.1093/molbev/msag235 (PMC13739495; doi:10.1093/molbev/msag235)
Supplement: Supplementary file 1 [file msag235_supplementary_data.pdf]

S1 Conditional probabilities

In section 2.2 we defined the emission probabilities as if the M observations in a single window were independent values. Let us remember why it is not always the case. Let's come back to the example of Papuans which split from Eurasians at time T_1, while Eurasians split from Africans at time T_2. It is clear that most of the private variants in Papuans relative to Eurasians are a subset of the private variants in Papuans relative to Africans. It follows that the number of private variants in Papuan relative to Eurasians and the number of private variants in Papuan relative to Africans are not independent values and can be computed using conditional probabilities.

We will explain in this section how to deal with conditional probabilities in specific demographic models. This is the default behavior of DAIseg (when the demography is appropriate) and what we used for inference in real data. We will use conditional probabilities for demographies where one can sort the reference populations such that after sorting, for each ancestry x and reference populations i and j such that $i < j$ the separation time between reference population i and x is larger than the separation time between reference population j and x. This is in particular the case for demography 1, corresponding to Papuan populations. Indeed, the African reference population has a larger separation time with Papuan, Denisovan and Neanderthal compared to the Eurasian reference population. We call here separation time between two populations the latest point in time where the two populations can have a coalescent event. For example, the separation time between Denisovan and Europeans is equal to the split time between Neanderthal and Denisovan.

When this condition is met, we sort in decreasing order the reference populations of our model in function of their split times as described above. The separation time here being used as a first approximation or proxy for the mean coalescent time. For each window, we then compute the decreasing sequence $Y = (y_1, ..., y_M)$ such that y_i is the number of private variants found in the window relatively to the first i reference populations (after sorting). Let us note μ the mutation rate, $T^c_{x,i}$ the mean coalescent time between an ancestry x and reference population o_i,

$$\lambda_i^x = \begin{cases} \mu \cdot L \cdot (T_{x,i} - T_{x,i+1}), & \text{if } 1 \leq i < M \\ \mu \cdot L \cdot T_{x,M}, & \text{if } i = M \end{cases}$$

and

$$y_i' = \begin{cases} y_i - y_{i+1}, & \text{if } 1 \leq i < M \\ y_M, & \text{if } i = M \end{cases}$$

Where y_i' represents the number private variants which accumulated in our target population between times $T_{x,i}$ and $T_{x,i+1}$. Finally, let Z_i be a discrete random variable following a Poisson distribution of parameter λ_i^x, we then have:

$$P(Y|x) = \prod_{i=1}^{M} \mathbb{P}(Z_i = y_i')$$

For demography 1 (fig. 1), we applied DAIseg with emission probabilities defined as in section 2.2 of the main text and then with the conditional probabilities as defined here. The difference in accuracy is shown in table S1. Using conditional probabilities increases the F-score of the inference. By comparing these results to table 1, one can notice that running the model without conditional probabilities outputs scores extremely similar to hmmix. This should come to no surprise as results for hmmix are coming from running the HMM twice, independently.

S2 Simulations

We used msprime Kelleher et al. (2016) to simulate data.

For the first demographic model we simulated 20 Papuans, 150 Africans and 150 Europeans, using demographic parameters from Gravel et al. (2011) which are illustrated in figure 1 and are as follow:

	Emission probabilities					
	Independent			Conditional		
	Precision	Recall	F-score	Precision	Recall	F-score
Neanderthal	0.97	0.6	0.74	0.92	0.75	0.83
Denisovan	0.73	0.995	0.84	0.82	0.96	0.88
Archaic	0.97	0.996	0.98	0.96	0.996	0.98

Table S1: Precision and recall scores for DAIseg applied to the simulated data, using two different ways to define emission probabilities: independent (see section 2.2) and conditional (see section S1).

Split times (in generations): Archaic-Sapiens (30,000), Neanderthal-Denisovan (24,000), African-Eurasian (2,400) and Eurasian-Papuan (1700). Admixture times and proportions: Neanderthal $\rightarrow$ Eurasian (2,000; 3%), Denisovan $\rightarrow$ Papuan (1,500; 4%). Population sizes: Ancestral (5,000), Sapiens before Out Of Africa (5,000), Archaic before split Neanderthal-Denisovan (7,500), Neanderthal (7,500), Denisovan (7,500), African (27,122), Eurasian (22,000), Papuan (4,947).

For the second demographic model, we simulated 20 individuals from the Target population, 150 from Reference population 1 and 150 from Reference population 2. We took demographic parameters such that the Target population could represent native Americans who received admixture from African populations after the European colonization. The demographic parameters are illustrated in figure 2 and are as follow:

Split times: Neanderthal-Sapiens (30,000), Reference Population 1 - Reference Population 2 & Target (2,400), Reference Population 2 - Target (1,200). Admixture times and proportions: Archaic $\rightarrow$ Reference Population 2 & Target (2,000; 3%), Reference Population 1 $\rightarrow$ Target (40; 15%). Population sizes: Ancestral (5,000), Sapiens before Out Of Africa (5,000), Neanderthal (7,500), Reference Population 1 (27,122), Reference Population 2 (22,000), Target (20,000).

We use diploid genomes of size 250,000,000 bp, constant recombination rate $r = 1.2 \cdot 10^{-8}$ and mutation rate $\mu = 1.25 \cdot 10^{-8}$. Each experiment was repeated 40 times with different random seeds. The simulation file is available at:
https://github.com/LeoPlanche/DAIseg/tree/main/simulations/simulations.py

S3 Demographic parameters estimation

The model requires as an input an assumed demographic model, consisting of admixture time, admixture proportion, and split time between populations. While in simulations these parameters are known, in real life problems, they can only be first estimated. To test how the uncertainty in these demographic parameters might affect the performance of the inference, we reran for the first demographic model four new sets of simulations, exactly as described above, but with the demographic parameters given as input to the HMM being randomly modified within a ±10%, ±20%, ±50% and ±80% range from their true value. Running the decoding algorithm with these parameters, we observe a slight decrease in accuracy of the model, especially when the parameters are off-set within a ±80% range. We then run our EM-algorithm to estimate the model parameters and rerun the decoding algorithm with the new estimated parameters. We observe in that case no decrease in accuracy even when the parameters are initialized with values far from the demographic truth (up to ±80%).

The results are given in table S2.

Starting from these modified input parameters, the EM-algorithm jointly estimates the corresponding coalescent times. We show that even when the inputs are modified within a ±80% range from their true value, the EM algorithm does not fall into a local extremum and constantly estimates the true coalescent times (figure S1).

	No offset					
	Without EM estimation			With EM estimation		
	Precision	Recall	F-score	Precision	Recall	F-score
Neanderthal	0.92	0.75	0.83	0.93	0.74	0.82
Denisovan	0.82	0.96	0.88	0.83	0.95	0.89
Archaic	0.96	0.996	0.98	0.97	0.98	0.97

	Maximum offset: 10%					
	Without EM estimation			With EM estimation		
	Precision	Recall	F-score	Precision	Recall	F-score
Neanderthal	0.92	0.75	0.83	0.93	0.74	0.82
Denisovan	0.82	0.96	0.88	0.83	0.95	0.89
Archaic	0.96	0.996	0.98	0.97	0.98	0.97

	Maximum offset: 20%					
	Without EM estimation			With EM estimation		
	Precision	Recall	F-score	Precision	Recall	F-score
Neanderthal	0.92	0.74	0.82	0.93	0.74	0.82
Denisovan	0.82	0.96	0.88	0.83	0.95	0.89
Archaic	0.96	0.996	0.98	0.97	0.98	0.97

	Maximum offset: 50%					
	Without EM estimation			With EM estimation		
	Precision	Recall	F-score	Precision	Recall	F-score
Neanderthal	0.92	0.71	0.8	0.93	0.74	0.82
Denisovan	0.8	0.95	0.87	0.83	0.95	0.89
Archaic	0.96	0.994	0.98	0.97	0.98	0.97

	Maximum offset: 80%					
	Without EM estimation			With EM estimation		
	Precision	Recall	F-score	Precision	Recall	F-score
Neanderthal	0.93	0.65	0.77	0.93	0.74	0.82
Denisovan	0.76	0.95	0.84	0.83	0.95	0.89
Archaic	0.95	0.98	0.97	0.97	0.98	0.97

Table S2: We run the same simulations as described in demography 1 (section 3.1.1). For each iteration of the simulations, the demographic parameters given as input to the HMM are being randomly modified within a ±10%, ±20%, ±50% and ±80% range from their true value. We first run the model with the emission probabilities directly computed from these modified values. The largest effect is found on Neanderthal detection with F-Score decreasing from 0.83 to 0.77 when applying a random offset of ±80%. After estimating the demography parameters using our EM algorithm, the same accuracy is obtained regardless of the initialization of the parameters.

S4 EM implementation

We detail the EM implementation in the case of demography 1 (Figure 1). Contrary to the Baum–Welch algorithm, which is also an EM algorithm, we do not consider emissions probabilities as independent, but depending on the shared demographic parameters. In that case, our model is parametrized by coalescent times $T_2^c, T_4^c, T_5^c, T_6^c$ for emissions probabilities and admixture times T_3 and T_1 for transition probabilities. We assume that mutation and recombination rates are fixed.

We denote:

$$\lambda = (\lambda_2, \lambda_4, \lambda_5, \lambda_6) := \mu L \cdot (T_2^c, T_4^c, T_5^c, T_6^c).$$

To estimate λ we use an EM algorithm for this special parametrization of the HMM. The core of EM is to:

a) Compute the expected value of the complete-data log-likelihood $Q(\lambda, \lambda')$ over the latent variables q, given the current parameter estimate λ'.

b) Find critical point of $Q(\lambda, \lambda')$ in λ.

c) Repeat until convergence.

For K independent observation sequences: $O = \{O^{(1)}, O^{(2)}, \dots, O^{(K)}\}$, where $O^{(k)} = (O_1^{(k)}, \dots, O_{M_k}^{(k)})$, the expected complete-data log–likelihood is:

$$Q(\lambda, \lambda') = \sum_{k=1}^{K} \sum_{Q^{(k)}} P(Q^{(k)} \mid O^{(k)}, \lambda') \log P(O^{(k)}, Q^{(k)} \mid \lambda)$$

The joint likelihood $P(O^{(k)}, Q^{(k)} \mid \lambda)$ factorizes as:

$$P(O^{(k)}, Q^{(k)} \mid \lambda) = \pi_{q_1} \prod_{t=1}^{M_k - 1} a_{q_t q_{t-1}} \prod_{t=1}^{M_k} b_{q_t}(O_t^{(k)}),$$

where we use the standard notation π, a and b for initial, transition and emission probabilities Due to the properties of log function Q splits into three terms: $Q(\lambda, \lambda') = Q_\pi + Q_a + Q_b$ connected with π, b and a. As we want to estimate only coalescent parameters, then

$$Q(\lambda, \lambda') = Q_b = \sum_{k=1}^{K} \sum_{t=1}^{M_k} \sum_{i=1}^{3} P(q_t^{(k)} = i \mid O^{(k)}, \lambda') \log b_i(O_t^{(k)})$$

For observation $O_i^{(k)} = (i, j)$ emission probabilities are

$$\begin{cases} b_0(O_{ij}) = \frac{\lambda_2^i}{i!} e^{-\lambda_2} \frac{(\lambda_4 - \lambda_2)^{i-j}}{(i-j)!} e^{-(\lambda_4 - \lambda_2)} \\ b_1(O_{ij}) = \frac{\lambda_2^j}{j!} e^{-\lambda_2} \frac{(\lambda_6 - \lambda_2)^{i-j}}{(i-j)!} e^{-(\lambda_6 - \lambda_2)} \\ b_2(O_{ij}) = \frac{\lambda_5^j}{j!} e^{-\lambda_5} \frac{(\lambda_6 - \lambda_5)^{i-j}}{(i-j)!} e^{-(\lambda_6 - \lambda_5)} \end{cases}$$

By the direct computation $\frac{\partial}{\partial \lambda}$ we obtain four non-linear equations defining critical value of Q–function with coefficients in standard gamma–variables $\gamma_t^{(k)}$ representing the posterior probability of being in state i at time t, given the observed sequence of emissions and the HMM parameters λ'

$$\gamma_t^{(k)}(i) = P(q_t^{(k)} = i \mid O^{(k)}, \lambda')$$

We use $fsolve$ function within the scipy.optimize module in Python to find the roots of a system of non-linear equations and update λ.

S5 Similarity to archaic genomes

To produce contour plots (Figure 4, Figure S3), the similarity for a segment is computed as such: we keep all the positions with an archaic private variant i.e. a variant that is found in an archaic genome, but absent in Africa. Then, there is a match if the archaic and Papuan individual share the private allele at this position. The similarity is the proportion of matches along all the positions containing an archaic private variant.

More precisely, let $m_1, \dots, m_N$ be modern African haplotypes, a_1, a_2 - archaic reference haplotypes and p_1, p_2 - Papuan haplotypes. Define the following sets of variable positions for a tract

$$V = \{\text{position } i : \exists k : \forall j : a_k[i] \neq m_j[i]\}$$
$$S = \{i \in V : \exists k, l : p_l[i] = a_k[i] \wedge \forall j : a_k[i] \neq m_j[i]\}$$

Then similarity is simply the ratio of the number of positions in each of the two sets

$$\text{similarity} = \frac{|S|}{|V|}.$$

We picked this similarity measure to avoid using the exact same subset of positions that are used by the model, which could bring circularity. We note that different similarity measures can

be found in the literature. One other possible similarity is to keep all the positions with a Papuan private variant i.e. a variant that is found in the Papuan target, but absent in Africa. Then, as before, there is a match if the archaic and Papuan individual share the Papuan private allele at this position. The similarity we use here (archaic private) is the same as in Yermakovich et al. (2024), while Browning et al. (2018) uses a similarity based on S^* which uses a subset of Papuan private SNPs. Using the Papuan private similarity, out of all segments detected as Denisovan, 15 % of them become closer to Neanderthal as compared to the original similarity measure (Figure S5). With this Papuan private similarity, 25% of segments detected as Neanderthal and 11% of segments detected as Denisovan show a similarity lower than 0.2 to either Archaic genome. 31% of the segments detected as Denisovan are closer to Neanderthal and 6% of the segments detected as Neanderthal are closer to Denisovan. Using the Papuan private tends to decrease the similarity which makes sense since the distance between the introgressed segment and the outgroup population (Africans) is expected to be longer than the distance between the sequenced archaic humans and the outgroup population. This is because the introgressed segment continued to evolve within the recipient population.

Finally, to compare as closely as possible with the contour plots of Browning et al. (2018), we restricted attention to regions supported by both methods. We intersected the DAIseg Denisovan segments longer than 80 kb with the archaic segments reported by Browning et al. (2018), retaining the intersected segments. Match rates to the Denisovan and Neanderthal reference genomes were then computed on these intersections. We plot (Figure S6) their similarity to archaic genomes using the private positions and match/mismatch information directly given in Browning et al. (2018) maps (SPrime output). We notice that as in Browning et al. (2018) original contour plots, the signal for two waves of introgression disappear, meaning that the presence/absence of this signal is probably more due to the similarity measure used than the introgression detection method. We also notice that the similarity of these regions lies much closer to Denisovan than Neanderthal, even if the archaic segments in Browning et al. (2018) contain both Denisovan and Neanderthal segments, further confirming the ability of DAIseg to distinguish between the two.

S6 Data availability

S6.1 Method

The method is implemented in Python and is available for use at:
https://github.com/LeoPlanche/DAIseg

S6.2 Inferred segments

The inferred introgression map for archaic segments in Papuans is available at:
https://github.com/LeoPlanche/DAIseg/blob/main/src/IntrogressionMap
The file will be gradually completed as more data becomes available. The samples and archaic segments described in this article will be kept as they are.

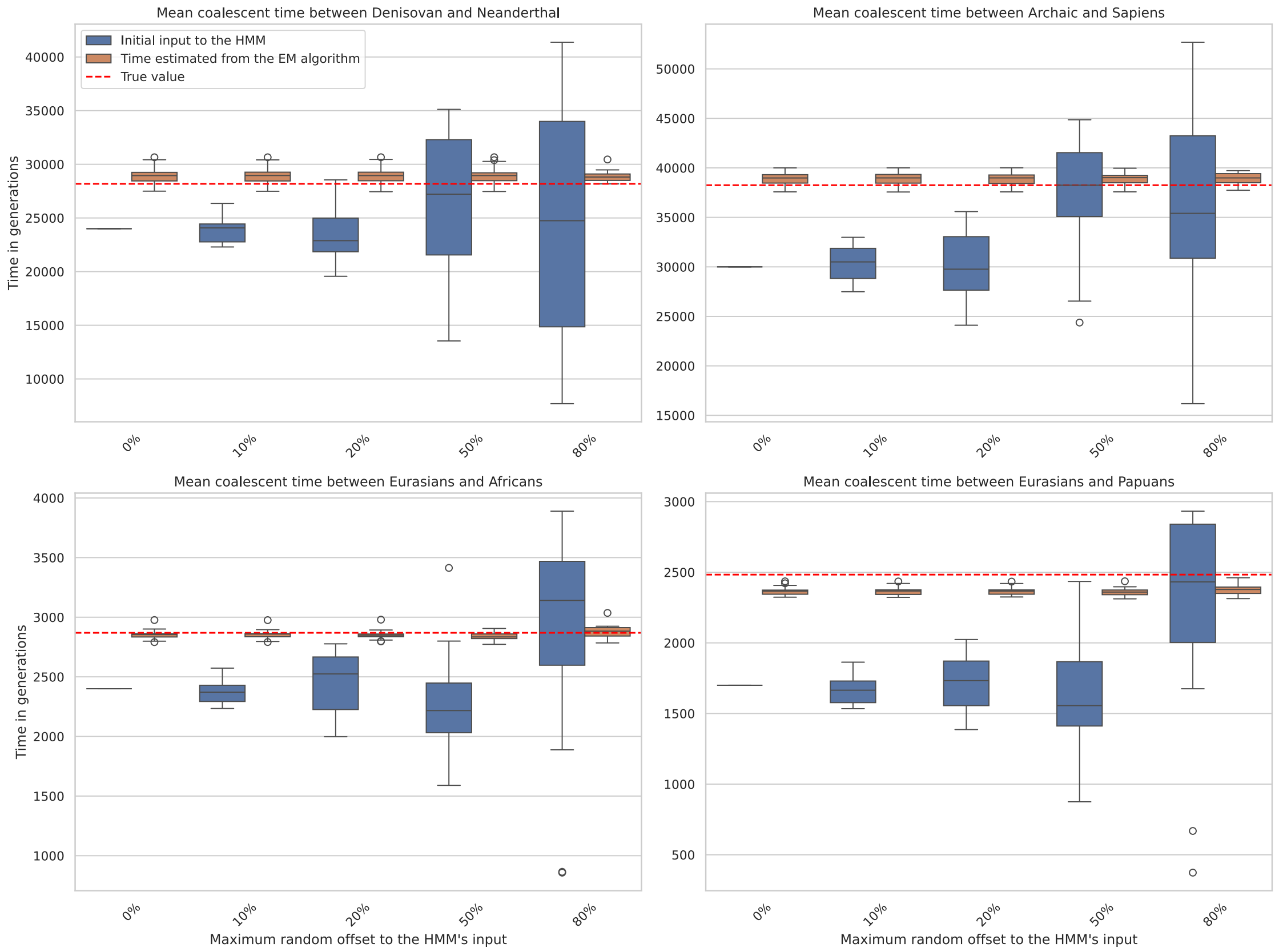

Figure S1: In simulations, joint estimations of the four demographic parameters involved in the emission probabilities for the first demography. On the x-axis, the maximum offset applied to the HMM's input. On the y-axis, the time in generations. In blue, the input values that are given to the HMM for each parameter, over 20 simulations. As the maximum offset gets bigger, the larger the variance between the different simulations. In orange, the parameter's estimate from the EM algorithm. In red, the true mean coalescent time, computed from msprime and averaged across all simulations.

S7 Supplementary figures

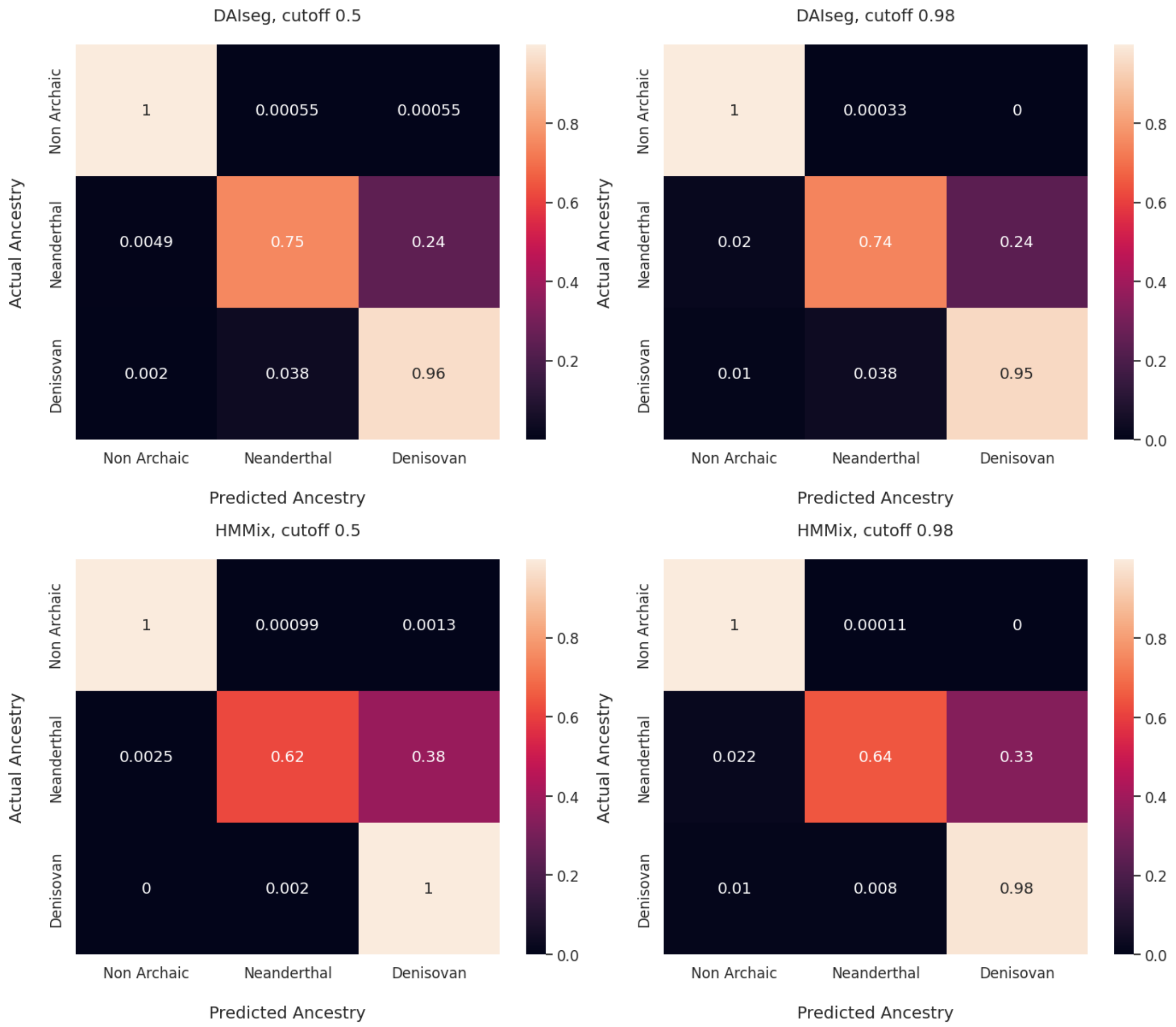

Figure S2: Confusion matrices for the ancestries inferred with DAIseg and hmmix based on simulations described in 3.1.1 with different posterior tresholds supporting archaic segments (0.5 and 0.98).

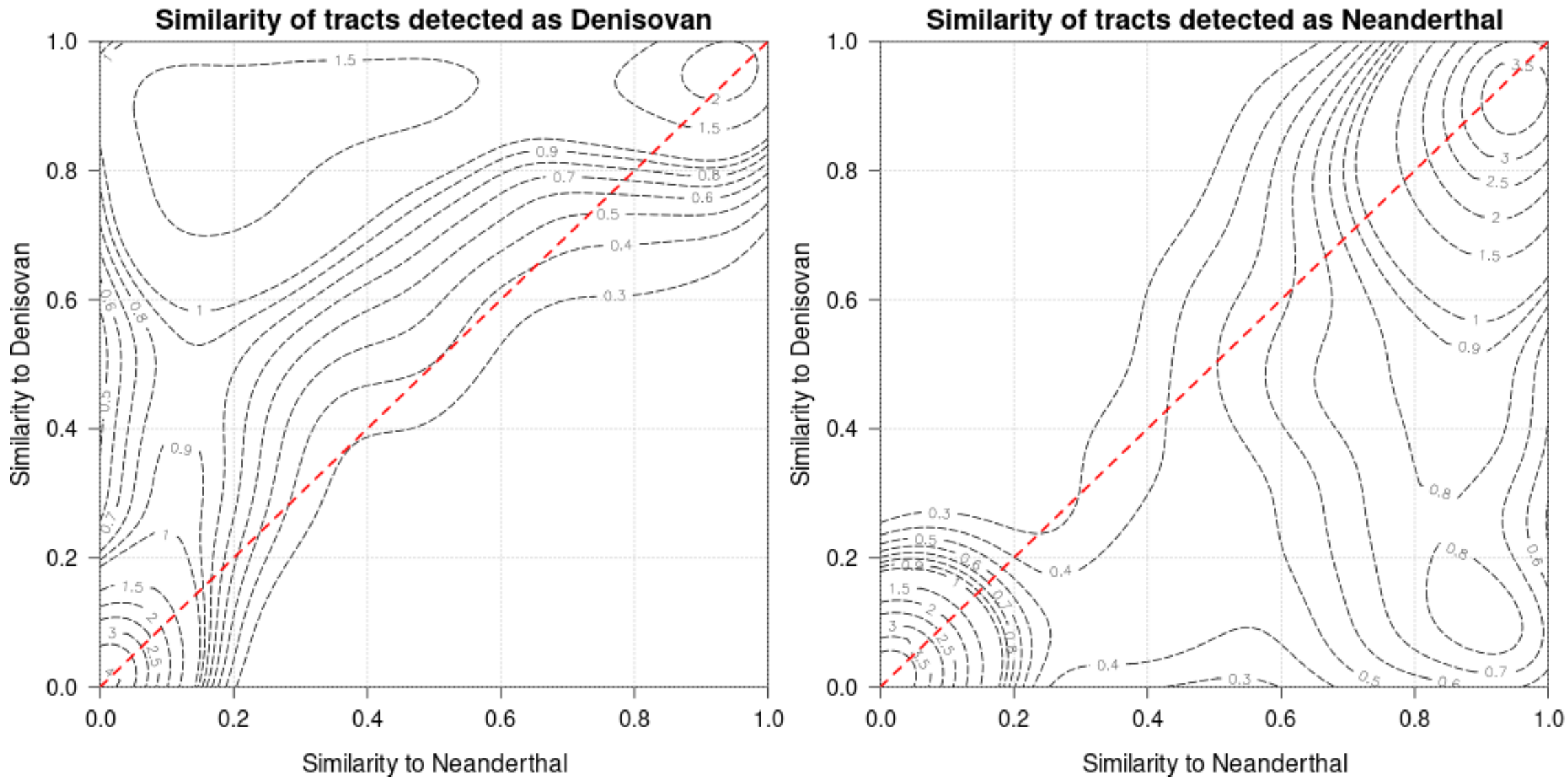

Figure S3: Measuring similarity to archaic genomes using the number of private variants in either Denisovan or Neanderthal. Segments, without any filtering on size, detected as Denisovan with DAIseg are shown in the left panel and those detected as Neanderthal in the right panel. The genetic similarity to the Vindija Neanderthal is shown on the x-axis and to the Denisovan on the y-axis. 96% of segments not detected as Archaic, 24% of segments detected as Neanderthal and 18% of segments detected as Denisovan shows a similarity lower than 0.2 to either Archaic genome. The red dashed line, $y = x$, corresponds to an equal similarity to the Neanderthal and Denisovan reference genomes.

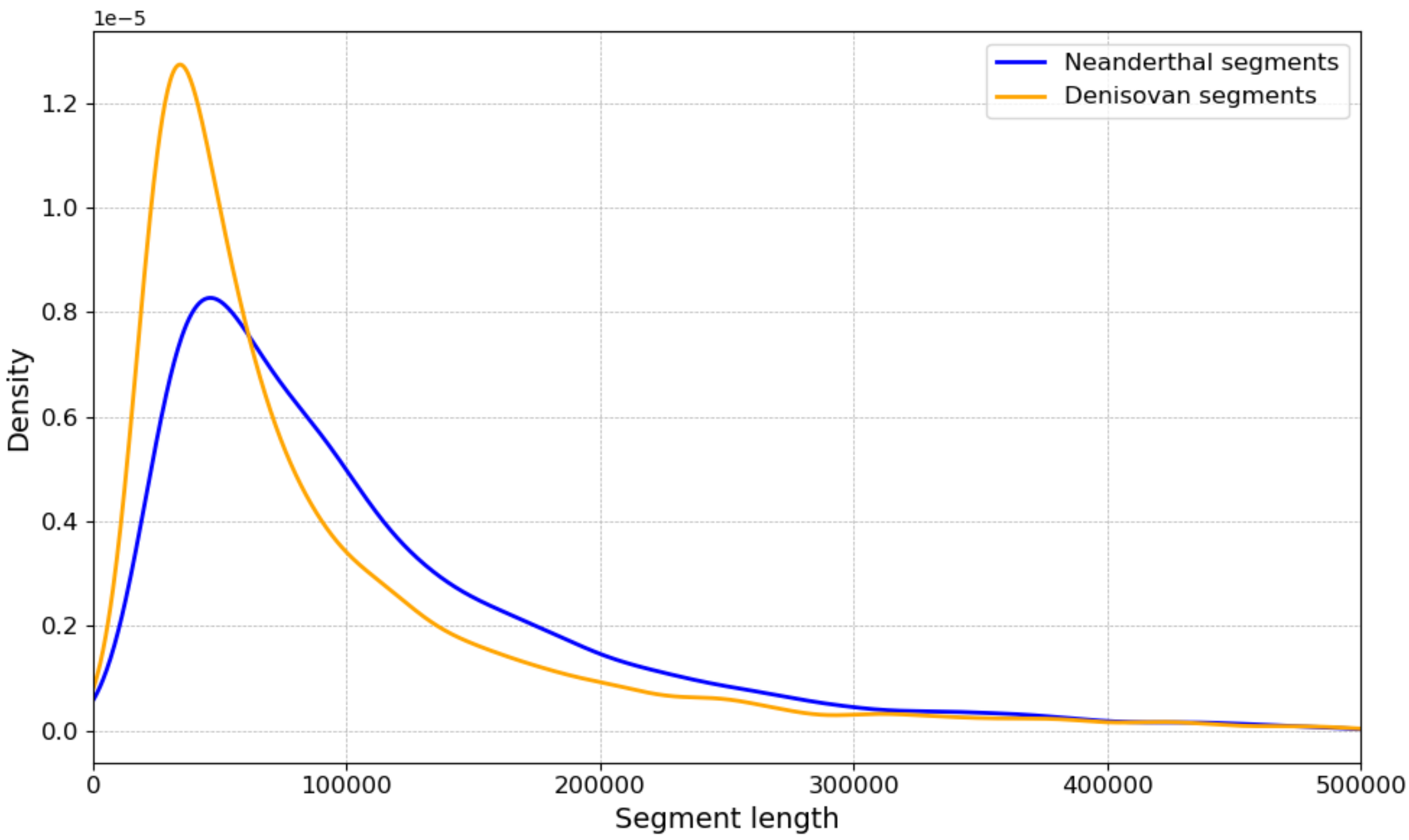

Figure S4: Denisovan segments are slightly shorter than Neanderthal segments. Average segment length is 109kb for Neanderthal and 87kb for Denisovan.

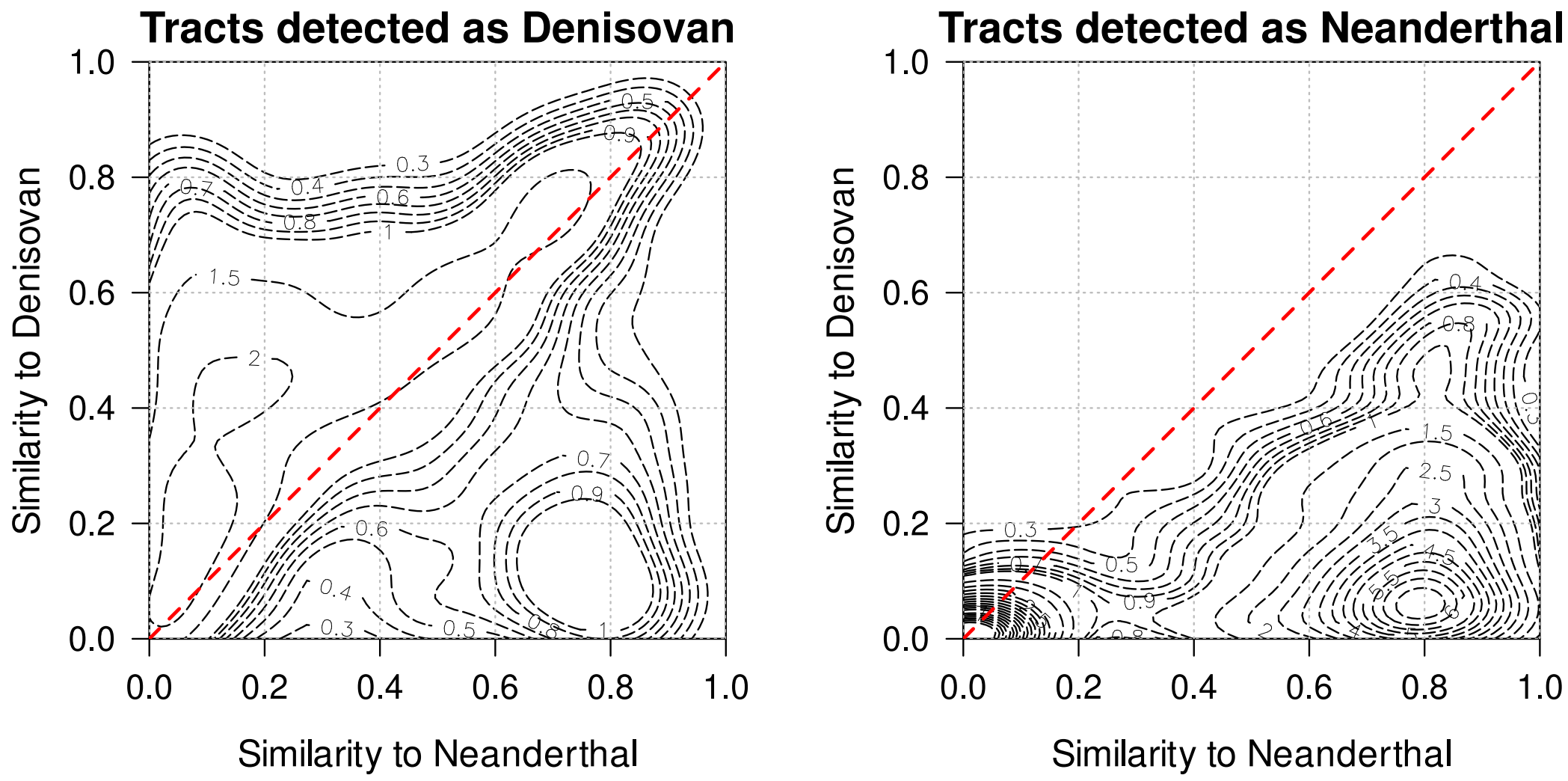

Figure S5: Measuring similarity to archaic genomes using the number of private variants in Papuans. Segments detected as Denisovan with DAIseg are shown in the left panel and those detected as Neanderthal in the right panel. The genetic similarity to the Vindija Neanderthal is shown on the x-axis and to the Denisovan on the y-axis. The similarity is defined using positions that are private to the Papuan target compared to the Yoruban outgroup. The red dashed line, y=x, corresponds to an equal similarity to the Neanderthal and Denisovan reference genomes.

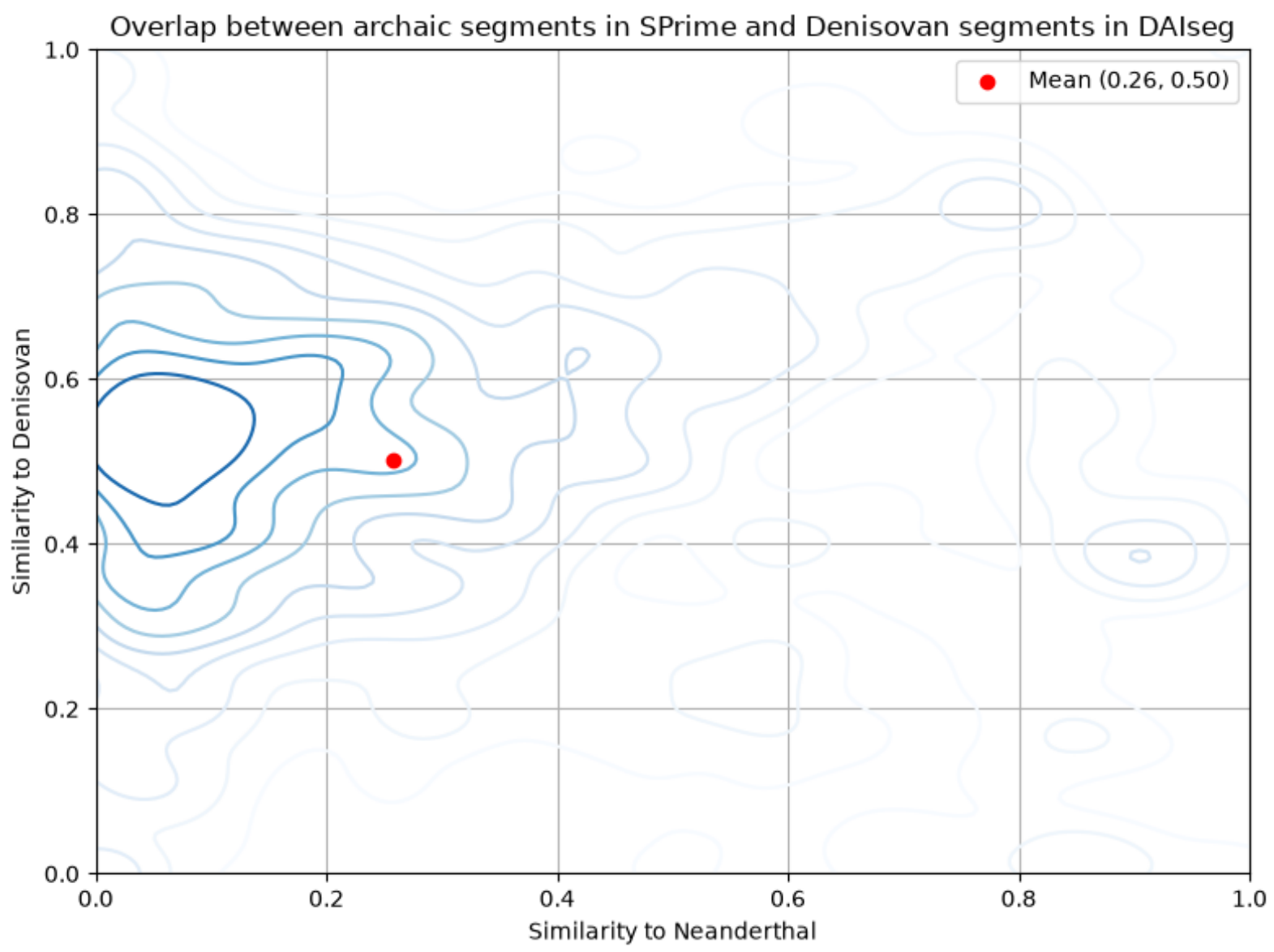

Figure S6: Similarity of regions that are detected as Denisovan in DAIseg and detected as archaic in Browning et al. (2018). The similarity is here computed directly using the private positions detected with SPrime that are given in Browning et al. (2018) maps.

References

Sharon R. Browning, Brian L. Browning, Ying Zhou, Serena Tucci, and Joshua M. Akey. Analysis of human sequence data reveals two pulses of archaic denisovan admixture. *Cell*, 173(1):53–61.e9, March 2018. ISSN 0092-8674. doi: 10.1016/j.cell.2018.02.031.

Simon Gravel, Brenna Henn, Ryan Gutenkunst, Amit Indap, Gabor Marth, Andrew Clark, Fw Yu, Richard Gibbs, Carlos Bustamante, David Altshuler, Richard Durbin, Goncalo Abecasis, David Bentley, Aravinda Chakravarti, Francis Collins, Francisco De La Vega, Peter Donnelly, Michael Egholm, Paul Flicek, and Jane Peterson. Demographic history and rare allele sharing among human populations. *Proceedings of the National Academy of Sciences*, 108:11983–11988, 07 2011. doi: 10.1073/pnas.1019276108.

Jerome Kelleher, A. Etheridge, and Gilean McVean. Efficient coalescent simulation and genealogical analysis for large sample sizes. *PLOS Computational Biology*, 12:e1004842, 05 2016. doi: 10.1371/journal.pcbi.1004842.

Danat Yermakovich, Mathilde André, Nicolas Brucato, Jason Kariwiga, Matthew Leavesley, Vasili Pankratov, Mayukh Mondal, François-Xavier Ricaut, and Michael Dannemann. Denisovan admixture facilitated environmental adaptation in Papua New Guinean populations. *Proceedings of the National Academy of Science*, 121(26):e2405889121, June 2024. doi: 10.1073/pnas.2405889121.
